# New Production Regulates Export Stoichiometry in the Ocean

**DOI:** 10.1371/journal.pone.0054027

**Published:** 2013-01-16

**Authors:** Tobias Tamelander, Marit Reigstad, Kalle Olli, Dag Slagstad, Paul Wassmann

**Affiliations:** 1 Department of Arctic and Marine Biology, University of Tromsø, Tromsø, Norway; 2 Institute of Ecology and Earth Sciences, University of Tartu, Tartu, Estonia; 3 SINTEF Fisheries and Aquaculture, Trondheim, Norway; Institute of Marine Research, Norway

## Abstract

The proportion in which carbon and growth-limiting nutrients are exported from the oceans’ productive surface layer to the deep sea is a crucial parameter in models of the biological carbon pump. Based on >400 vertical flux observations of particulate organic carbon (POC) and nitrogen (PON) from the European Arctic Ocean we show the common assumption of constant C:N stoichiometry not to be met. Exported POC:PON ratios exceeded the classical Redfield atomic ratio of 6.625 in the entire region, with the largest deviation in the deep Central Arctic Ocean. In this part the mean exported POC:PON ratio of 9.7 (a:a) implies c. 40% higher carbon export compared to Redfield-based estimates. When spatially integrated, the potential POC export in the European Arctic was 10–30% higher than suggested by calculations based on constant POC:PON ratios. We further demonstrate that the exported POC:PON ratio varies regionally in relation to nitrate-based new production over geographical scales that range from the Arctic to the subtropics, being highest in the least productive oligotrophic Central Arctic Ocean and subtropical gyres. Accounting for variations in export stoichiometry among systems of different productivity will improve the ability of models to resolve regional patterns in carbon export and, hence, the oceans’ contribution to the global carbon cycle will be predicted more accurately.

## Introduction

The concept of new vs. regenerated primary production *sensu* Dugdale and Goering (1967) [1] has profoundly shaped the understanding of biological production and carbon sequestration in the ocean. The revelation that new production based on allochthonous nutrients supplied to the surface layer approximates export of organic matter to the deep ocean [2] has been widely adopted as a law of nature in ecosystem models used to quantify the contribution of the oceans’ biological carbon pump to the global carbon cycle. While export from a steady-state system must be balanced by an equivalently large input of new nutrients, recycling of carbon and growth-limiting nutrients by the pelagic food web profoundly impacts the potential of a system to export carbon [3], [4]. Because organic nitrogen is remineralised faster than carbon in large parts of the ocean [5], the common assumption of constant elemental stoichiometry (e.g. Redfield) for converting nitrogen export to carbon typically underestimates the strength of the biological carbon pump and therefore carbon export (e.g. [6]). Assuming that C:N is constant over spatially heterogeneous environments also constrains the ability of models to resolve regional patterns in carbon export, which are crucial for understanding ecosystem functioning. Model experiments with a depth-dependent particulate organic C:N ratio significantly increased carbon sequestration compared to a constant ratio model [7]. Furthermore, carbon overconsumption in response to increased atmospheric CO_2_ is suggested to increase the spread of suboxic regions in the ocean due to respiration of excess carbon exported from the surface layer [8]. These findings suggest that our ability to predict how perturbations to the ocean environment affect the marine ecosystem in part depend on our understanding of the stoichiometric coupling between carbon and nutrients. At present, knowledge of the regulation of exported C:N is limited which poses a constraint to further development of ecosystems models. Because nitrogen recycling within the euphotic zone varies regionally with primary production [2], export stoichiometry and, hence, the efficiency of the biological carbon pump should reflect nutrient availability or trophic state (oligo-, meso-, eutrophy) of pelagic ecosystems.

We use published and unpublished data on vertical flux of particulate organic carbon (POC) and nitrogen (PON) from the European Arctic Ocean to test the hypothesis of constant export stoichiometry. The same methodology was adopted in all studies in this region, which allows comparisons of the data among regions in order to determine spatial patterns. The Arctic Ocean was also not included in a previous synthesis of export stoichiometry [9]. Furthermore, we assess the anticipated relationship between nitrate-based new production as a measure of trophic state and export stoichiometry over large geographical scale based on published data from other primarily nitrogen-limited parts of the World Ocean.

## Materials and Methods

### Data Collection Analytical Methods

**Figure 1:**
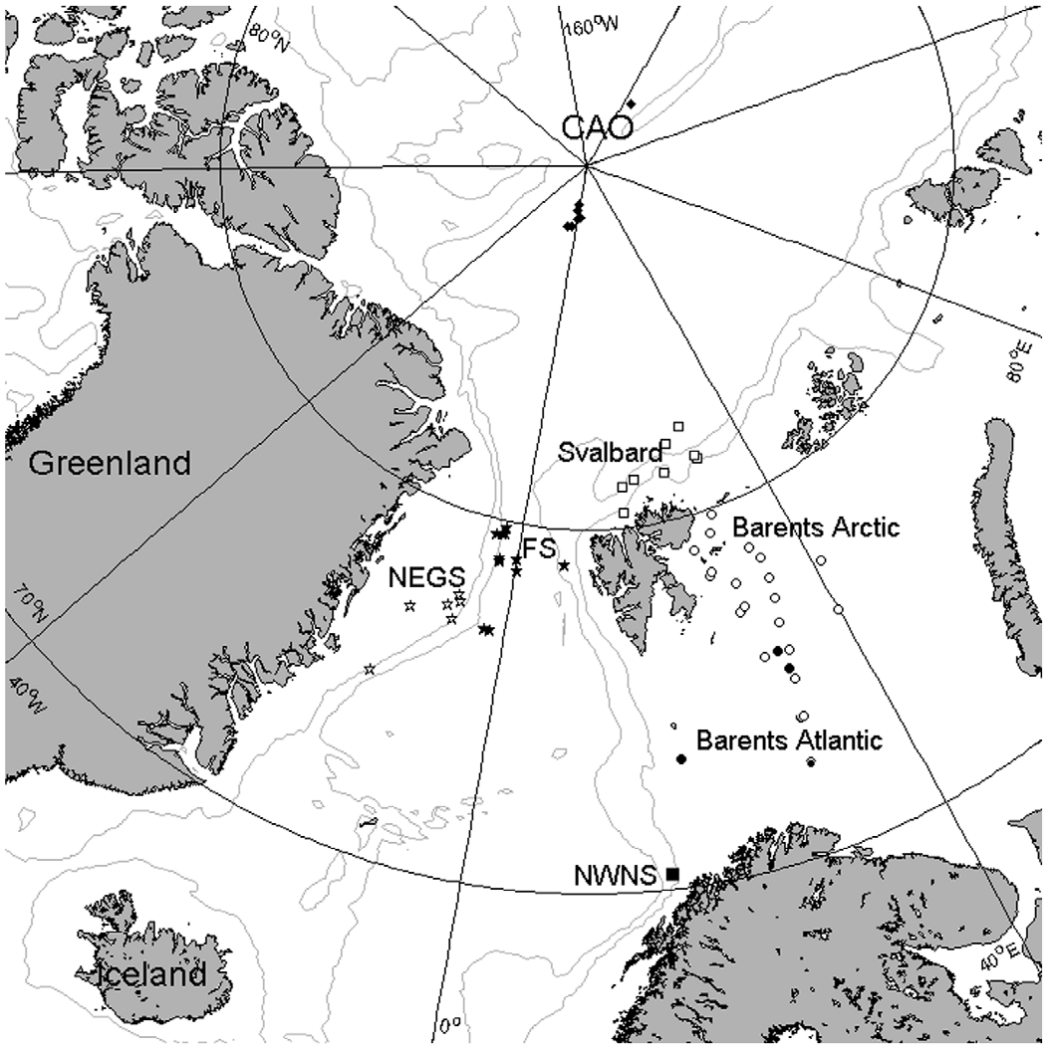
Map of the European Arctic with locations of sediment trap deployments indicated according to the defined sub-regions: Central Arctic Ocean (CAO, black diamonds), Fram Strait (FS, black pentagrams), NE Greenland Shelf (NEGS, white pentagrams), Svalbard shelf break (Svalbard, white squares), Barents Sea Arctic (Barents Arctic, white circles), Barents Sea Atlantic (Barents Atlantic, black circles), and NW Norwegian shelf (NWNS, black square). Locations visited more than once have overlapping symbols. The NWNS was visited 6 times in the same year. Bathymetry (grey lines) is indicated by the 500 and 2000 m isobaths.

Vertical export in the European Arctic Ocean was determined by means of short-term deployed surface-tethered drifting sediment traps at a total of 64 stations or occasions (Fig. 1). Between 1 and 14 sediment traps were used to collect sinking particulate matter in the 20–200 m depth range. A typical array consisted of traps situated at 20, 30, 40, 50, 60, 90, 120 and 200 m depth, and was attached to a surface-tethered float or a drifting ice floe and deployed for circa 24 h without preservatives. The sediment traps consisted of duplicate parallel cylinders (height:diameter ratio 6.25) mounted in a gimballed frame equipped with a vane. The cylinders maintain vertical orientation perpendicular to the current direction at the moderate current velocities prevailing under all deployments [10]. The trapping efficiency for particulate organic carbon is 90–110%, determined by comparison with the ^234^Thorium method, and the traps therefore provide reliable estimates of particle fluxes [10].The observations were *a priori* grouped into seven sub-regions based on the dominant water mass, ice conditions, and bottom depth (Table 1). The observations are temporally constrained to the productive season (March-August), but the temporal resolution and distribution of observations over the season vary among regions.

**Table 1:** Characteristics of sub-regions of the European Artic Ocean.

Region	Stations (n)	Bottom depth (m)	Ice type	Water masses	Origin of data
Central Arctic Ocean	7	2500–4000	MYI	ArW, AtW (below 250 m)	[25]
Svalbard shelf break	8	300–3700	FYI	ArW, SMW, mAtW (below 50 m)	[18], [26], [27], K.O. unpubl
Fram Strait	11	1000–3200	FYI	ArW, AtW	[28], M.R. unpubl
NE Greenland shelf	6	250–310	FYI	ArW, SMW, AtW (below 100 m)	M.R. unpubl
Barents Sea Arctic	19	170–350	FYI	ArW, AtW (in trenches)	[11], [18], [26]
Barents Sea Atlantic	7	220–480	None	AtW	[11], [26]
NW Norwegian shelf	6	500	None	AtW, NCW	[29]

Ice types are multi-year ice (MYI) and first-year ice (FYI). Water masses are Arctic Water (ArW), Atlantic Water (AtW), Surface Melt Water (SMW), modified Atlantic Water (cooled and diluted; mAtW), and Norwegian Coastal Water (NCW), as defined by Sundfjord et al. 2007 [13].

Particulate organic carbon (POC) and nitrogen (PON) were determined in GF/F filter samples of the sediment trap content using an elemental CHN analyser. The filters were dried and exposed to HCl fumes in order to remove inorganic carbon prior to analyses [11]. POC and PON values were corrected for background contamination determined in blank filters. POC and PON fluxes in units of mass were converted to molar unit using the atomic weights of C and N.

Exported POC:PON and new production from other parts of the World Ocean were obtained from the literature. Only observations from the upper 500 m of the water column were included in order to allow comparisons with the data from the Arctic Ocean (Table 2). New production in the European Arctic Ocean was simulated [6] and published values from other regions were mostly based on nitrate inventories (Table 2). Data on new production were in most cases available in units of carbon based on conversion from nitrate uptake to carbon using either observed DIC:NO_3_ drawdown ratios or Redfield C:N. Where new production was available only in units of nitrogen (W Sargasso Sea), it was converted to carbon using the Redfield C:N ratio.

**Table 2:** Mean exported POC:PON ratio (mean±SD (SE)) of particulate organic matter sampled by means of sediment traps, and mean annual new production (NP) in regions included in Fig. 4, with reference to source.

Region, abbreviation	Obs	Trap depth	POC:PON	Comments	Ref POC:PON	NP	NP method	Ref NP
	(n)	(m)	(a:a)			(mol C m^−2^ yr^−1^)		
Central Arctic Ocean, CAO	54	20–200	9.7±1.5 (0.2)	August	This study	1.0	Model; 19-year mean	This study
Svalbard shelf break, Svalbard	31	20–200	7.7±1.5 (0.3)	May, July	This study	2.4	Model; 19-year mean	This study
Fram Strait, FS	86	20–200	7.7±2.3 (0.2)	April-June, August	This study	5.8	Model; 19-year mean	This study
NE Greenland shelf, NEGS	48	20–200	8.9±2.0 (0.3)	April-May	This study	4.2	Model; 19-year mean	This study
Barents Sea Arctic, BSAr	136	20–200	8.5±1.8 (0.2)	March, May, July	This study	4.2	Model	[26]
Barents Sea Atlantic, BSAt	54	20–200	7.4±1.0 (0.1)	March, May, June, July	This study	6.7	Model	[26]
NW Norwegian shelf, NWNS	73	20–200	8.6±1.6 (0.2)	April-September	This study	6.6	Model	[30]
Northeast Water Polynya, NEW		130	9.2	Annual cycle	[31]	2.7		[32]
North Open Water Polynya, NOW	12	200–500	8.5±0.7 (0.2)	Annual cycle	[33]	6.4	NO_3_ inventory	[34]
E Greenland shelf, EGS	20	245	9.9±1.2 (0.3)	Annual cycle	[35]	3.3		[32]
East Beaufort Sea, EBS	6	100, 200	9.3±1.3 (0.5)	3 years	[36]	1.2	NO_3_ inventory	[37]
N Pacific subarctic gyre, K2	19	150–500	8.1±0.5 (0.1)	July-August	[38]	4.0	NO_3_ inventory	[39]
Iberian upwelling, IU	50	30–200	8.0±0.8 (0.1)	10 times, August	[40]	9.3	NO_3_ inventory	[41]
NE Atlantic subtropical gyre, ESTOC	3	200–500	10.2	6 times in 2 years NP is mean of same years as CN export	[42]	0.8	NO_3_ inventory	[42]
N Pacific subtropical gyre, HOT	206	150–500	10.2±0.3	Monthly, 11 years	[9]	1.4	O_2_ prod, NP is 50% of primary production	[43]
NE Subarctic Pacific, OSP	5	100–500	8.0±1.4 (0.5)	May, 5 times	[21]	2.2	NO_3_ inventory	[44]
W Sargasso Sea, BATS		500	7.8±1.4	9 years, biweekly sampling	[45]	2.5	NO_3_ inventory. Conv. to C by Redfield C:N	[46]

### Statistical Analyses

Exported POC:PON data were not normally distributed wherefore regional variations and deviations from Redfield were assessed by a nonparametric Kruskal-Wallis test with the Multiple Comparisons post hoc test in Statistica® and a Wilcoxon signed rank test in R (www.r-project.org), respectively. Linearity of the POC:PON ratio was assessed by the type two regression Standard Major Axis of log-transformed POC on log-transformed PON flux using the package lmodel2 in R because the variation was uncontrolled in both directions, and to overcome the bias associated with ordinary least square regression when testing the slope against a given value (in this case 1) [12]. In log-log space, a slope equal to one implies that C and N occur in the same proportions across the whole range of C and N export values, and the SMA regression was used to test this hypothesis, following Sterner et al. (2008) [12]. Slopes were considered to be significantly different from one, indicating deviation from constancy, when the 95% confidence interval did not include the value one. The relationship between mean POC:PON and new production was assessed by a linear regression following assessments of normal distributions (Shapiro-Wilk tests, P = 0.69 and P = 0.39, respectively). The Svalbard shelf break area was excluded from the regression due to strong advection of organic matter reflective of a more southerly Atlantic production regime into this area by the West Spitsbergen Current [13], [14], material that contributes to vertical export in addition to autochthonous production.

## Results and Discussion

### Regional Patterns in the European Arctic Ocean

**Figure 2:**
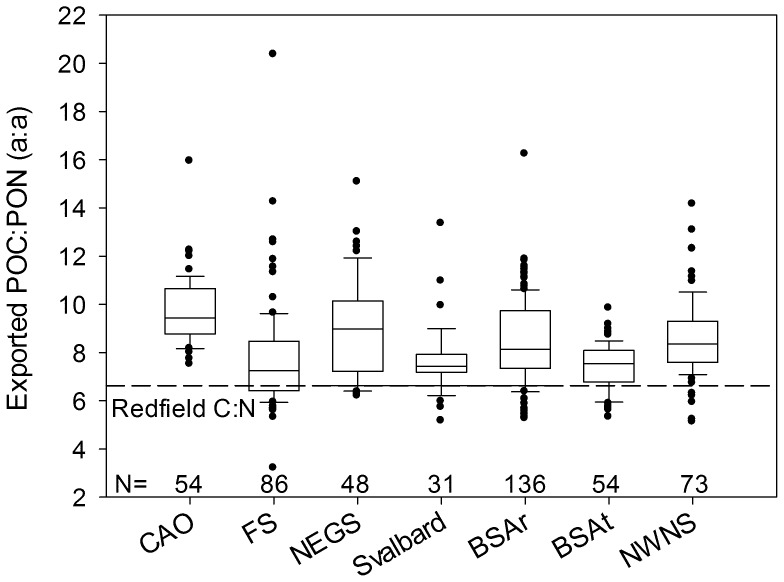
Ratio between vertically exported POC and PON (a:a) in different parts of the European Arctic Ocean, with number of observations in each region. The line indicates the median, boxes and whiskers are the 25^th^ and 75^th^ percentiles and 10^th^ and 90^th^ percentiles, respectively, and dots are outliers. The dashed line indicates the Redfield C:N ratio. Abbreviations are CAO – Central Arctic Ocean, FS – Fram Strait, NEGS – NE Greenland Shelf, Svalbard – Svalbard shelf break, BSAr – Barents Sea Arctic, BSAt – Barents Sea Atlantic, NWNS – NW Norwegian shelf.

The exported POC:PON ratio increased with depth in only 8 of 61 vertical flux profiles (not shown), and comparisons are therefore based on all observations between 20 and 200 m depth. The exported POC:PON ratio was significantly different among regions (Kruskal-Wallis ANOVA, P<0.0001, N = 482, Fig. 2) and was higher in the Central Arctic Ocean than elsewhere (P<0.01, Multiple Comparisons post hoc test) except over the NE Greenland shelf (P = 0.062). Variations were significant over short spatial scales relating to geophysical boundaries shown by the higher POC:PON ratio in the seasonally ice-covered northern Barents Sea than in the southern part dominated by warmer (>0°) Atlantic Water (P = 0.002). This pattern corresponds to the higher abundance of diatoms and flagellates (*Phaeocystis* sp.) in the phytoplankton communities of the Arctic and Atlantic sections, respectively [11]. Diatoms are exported to greater extent than *Phaeocystis* sp. [15] and represent a carbon-rich fraction of the exported organic matter likely owing to their high lipid content. Seasonal biases in the data, e.g. towards pre-bloom conditions in late winter in the Fram Strait and over the NE Greenland shelf and towards summer in the Central Arctic Ocean, could further increase the differences among regions.

The mean exported POC:PON was above Redfield (6.625, a:a) in all seven regions (Wilcoxon signed rank test, P<0.0001 in all cases). Conversion of nitrogen-based export estimates to POC using the Redfield ratio therefore underestimates carbon export in the European Arctic Ocean, consistent with most other oceanic regions [9]. The error is highest in the Central Arctic Ocean where the mean exported POC:PON of 9.7 implies that POC export is c. 40% (i.e. 9.7/6.6) higher than Redfield-based estimates. In order to estimate the impact of variable stoichiometry on annual POC export in the European Arctic Ocean, potential POC export was calculated by multiplying spatially integrated annual new production by the POC:PON ratio of the exported material. The potential POC export is 208 Tg C yr^−1^ for the entire area when using the observed POC:PON ratios specific to each region (“Observed Variable C:N”, Table 3). This is almost 30% higher than potential POC export when applying the Redfield C:N ratio (165 Tg C yr^−1^), and c. 10% higher than estimates based on a constant C:N ratio of 7.6 as used in the model described in Wassmann et al. 2006 [6]. Regional variations in the stoichiometry of exported organic matter therefore significantly impacts modelled POC export, and accounting for this variation is an improvement from constant stoichiometry models. However, these estimates would benefit further from more data from the Central Arctic Ocean which has relatively few observations and which also represents the largest area.

**Table 3:** Spatially integrated potential export of particulate organic carbon in Tg C yr^−1^ in the European Arctic Ocean (excluding the NW Norwegian shelf) calculated based on different C:N ratios.

		C:N ratio		
Region	Area 10^3^ km^2^	Redfield	Observed Constant	Observed Variable
Central Arctic Ocean	4489^a^	48.5	55.7	71.1
Fram Strait	172^b^	10.4	11.9	12.2
NE Greenland Shelf	305^c^	13.5	15.4	18.2
Svalbard	6^a^	0.1	0.2	0.2
Barents Sea Arctic	500^d^	21.8	25.0	27.9
Barents Sea Atlantic	1012^e^	70.6	81.0	79.3
Total	6484	164.9	189.2	208.8

Redfield C:N is 6.625 and Observed Constant C:N is 7.6 [6]. For Observed Variable, see Table 2.

a From Jakobsson et al. 2004 [47].

b Area between 76° and 80° N deeper than 500 m.

c Area between 70° and 82° N shallower than 500 m.

d Assumed equal to average maximum extent of sea ice [48].

e Total area of Barents Sea from Jakobsson et al. 2004 [47] minus Barents Sea Arctic (d).

### Assessment of Linearity of Export Stoichiometry

**Figure 3:**
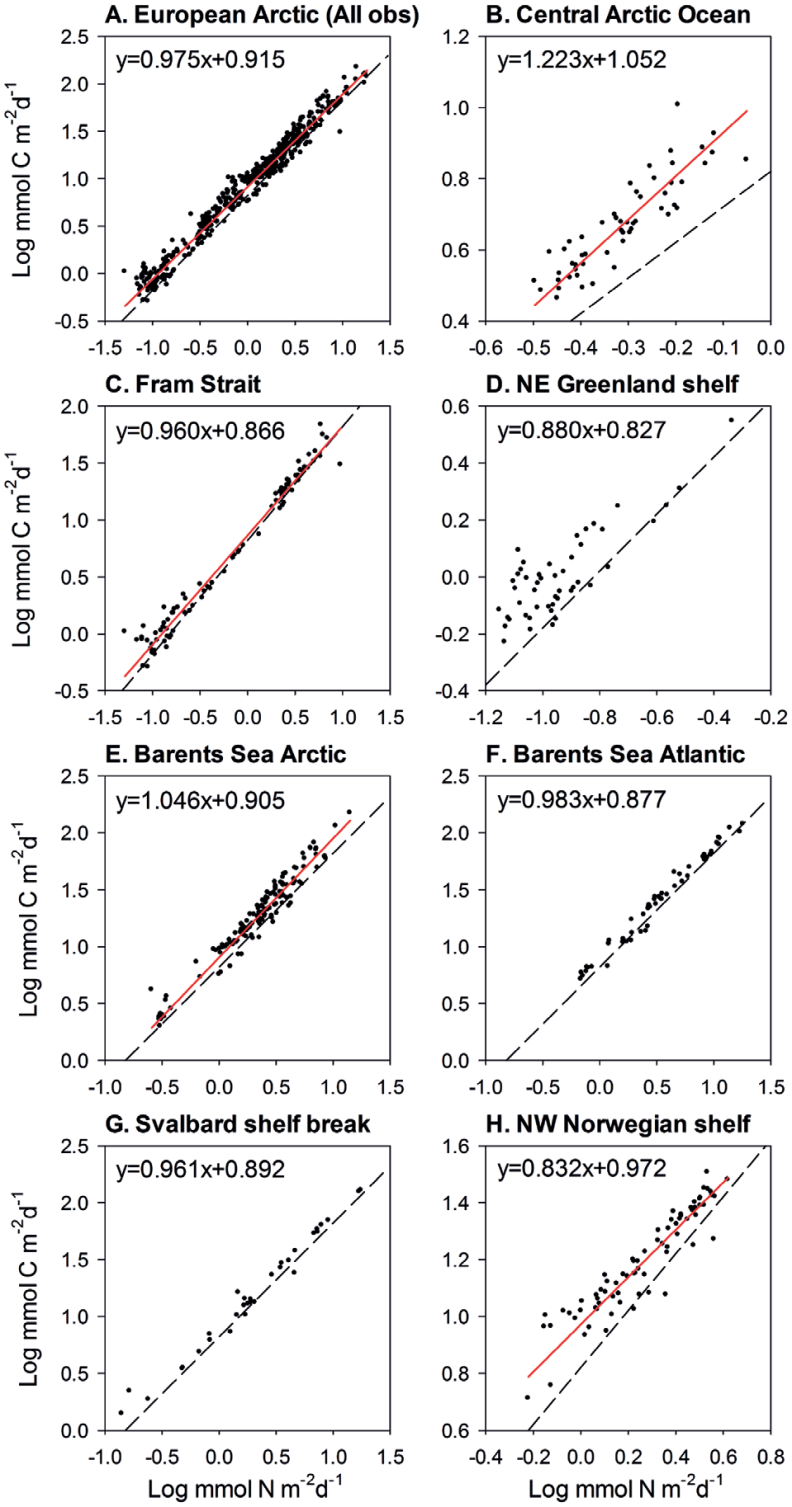
Relationships between vertical flux of POC and PON in the European Arctic Ocean. Log-log plots of POC on PON (log mmol m^−2^ d^−1^) and Standard Major Axis regressions based on all data (A) and for each sub-region (B–H). Regressions with slope significantly different from one are indicated by red lines. The stippled line indicates the Redfield C:N ratio. Regression coefficients with confidence intervals are given in Table 4.

Linearity of the exported POC:PON ratio was assessed by determining the Standard Major Axis (SMA) regression of log-transformed POC flux on log-transformed PON flux, and testing the null hypothesis slope = 1 (i.e. constant ratio across the range of observed POC and PON fluxes). The SMA slope for the entire dataset was significantly smaller than one (slope = 0.975, 95% C.I. = 0.961–0.989, N = 482), implying that the POC:PON ratio is lower when the flux is high compared to when the flux is low. Hence, the POC:PON ratio decreases with increasing vertical flux (Fig. 3A, detailed regression results in Table 4). This suggests that nitrogen recycling is weaker during high-flux events corresponding to spring-bloom conditions prior to nitrogen depletion compared to low-flux events when detrital material likely comprises a larger fraction of the exported material. Among individual regions, the NW Norwegian Shelf revealed a depletion of POC relative to PON (slope = 0.832, Fig. 3H) whereas an equally strong enrichment was observed in the Central Arctic Ocean (slope = 1.22, Fig. 3B). In three regions the exported POC:PON ratio was constant (i.e. SMA slope not significantly different from one). Factors acting at regional scale therefore contribute to shaping export stoichiometry. In addition to variations in the relative contribution of dominant phytoplankton taxa (diatoms and flagellates, see above) among regions, organic matter released from sea ice represent another carbon-rich fraction of vertically exported material in ice-covered areas [16]–[18] that could partially explain the enrichemnt in POC over PON in the Central Arctic Ocean and northern Barents Sea. Colonisation of sinking particles by bacteria and heterotrophic flagellates, which was observed in a temperate upwelling system, would have an opposite effect by increasing the nitrogen content of initially nitrogen-deprived organic matter [39]. The same process could occur in the Arctic Ocean as well, but this remains speculative until more detailed data become available. In combination with dissolution and degradation of particulate organic matter, these factors contribute to shaping elemental fluxes from the water column and, therefore, to the net carbon export efficiency in terms of POC:PON stoichiometry.

**Table 4:** Results of Standard Major Axis regressions of log-POC flux on log-PON flux.

Region	N	Slope	Intercept
European Arctic – all observations	482	0.975 (0.961–0.989)	0.915 (0.914–0.915)
Central Arctic Ocean	54	1.223 (1.077–1.390)	1.052 (1.007–1.104)
Fram Strait	86	0.960 (0.927–0.993)	0.866 (0.860–0.873)
NE Greenland shelf	48	0.880 (0.745–1.038)	0.827 (0.701–0.977)
Barents Sea Arctic	136	1.046 (1.006–1.087)	0.905 (0.892–0.918)
Barents Sea Atlantic	54	0.983 (0.945–1.024)	0.877 (0.855–0.898)
Svalbard	31	0.961 (0.912–1.012)	0.892 (0.876–0.907)
NW Norwegian Shelf	73	0.832 (0.764–0.907)	0.972 (0.953–0.990)

Regression slope and intercept with 95% confidence intervals in brackets.

### Global Patterns and Regulation of Export Stoichiometry

**Figure 4:**
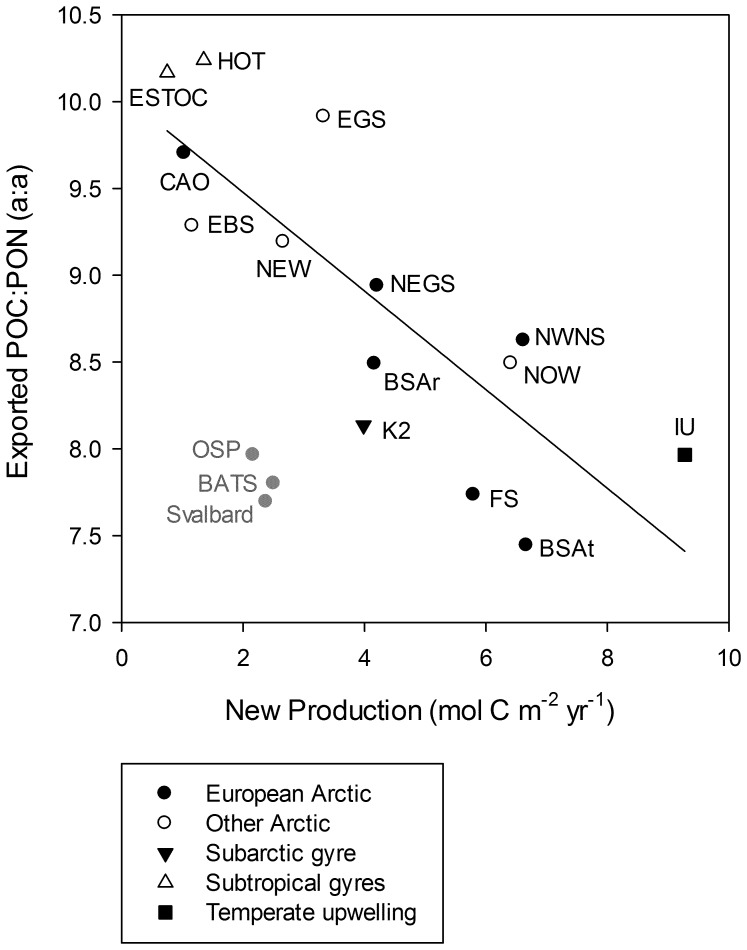
Mean exported POC:PON ratio for each region against average annual new production. The regression (y = −0.2840x+10.0450, N = 14) is significant (P = 0.0004) with an R^2^ of 0.66. The Svalbard shelf break was excluded from the regression due to its advective nature (see Methods). See Table 2 for abbreviations for individual sites. Regions shown for comparison but which are not included in the regression (in grey) are Svalbard, NE subarctic Pacific (OSP) and W Sargasso Sea (BATS).

From a modelling point of view where regional variations in carbon export integrated over time is of primary interest, a unifying concept for regulation of export stoichiometry and, hence, carbon export efficiency, is desirable. We tested the hypotheses that nitrate-based new production is a driver of export stoichiometry across systems characterised by different trophic state (Table 2). A linear regression of the mean exported POC:PON ratio in each region on new production revealed a significant negative relationship (R^2^ = 0.66, P = 0.0004, Fig. 4), with exported POC:PON decreasing by 0.28 per mole carbon produced based on nitrate uptake. Standard errors of the slope and intercept were 0.0585 and 0.2811, respectively. Albeit the variation in exported POC:PON is high (range of ca 1) for any new production rate, the regression suggests stronger nitrogen recycling in nutrient-poor oligotrophic environments, resulting in higher carbon export per unit limiting nutrient as compared to more nutrient-rich mesotrophic environments. More efficient nitrogen recycling under oligotrophic regions is governed by the pelagic food web structure, which is characterized by an “inverted biomass pyramid” (i.e. heterotrophic to autorophic biomass ratio >1), resulting in consumer-control of nutrient pools and flows [19], [20]. Under eutrophic conditions where autotrophs dominate, nitrogen recycling is less efficient which again favours export of the accumulated organic matter [20]. However, even in the most productive regions the C:N ratio of exported particulate organic matter exceeds the Redfield ratio (Table 2). The observed relationship is consistent with the overall increasing proportion of PON over POC with increasing vertical flux (Fig. 3A), since the highest fluxes typically are recorded during phytoplankton blooms when new production is highest. Regions where primary production is controlled by other factors than nitrate deviate from this pattern. This is exemplified in Fig. 4 by a High Nutrient Low Chlorophyll area of the subarctic North Pacific where nitrate never is depleted in the surface layer [21], and the W Sargasso Sea where phosphorus excerts stronger control of primary production than nitrogen [22]. Hence, exceptions to what appears to be a general dependence of exported POC:PON on new production are explained by different controls of primary production in these oceanographic regions, and dominance of other phytoplankton functional groups [23]. Since nitrate limits primary production in most parts of the world’s oceans [24], nitrate-based new production emerges as a driver of export stoichiometry across climatically-defined domains (arctic, temperate, tropics).

### Conclusions

This study shows that the C:N ratio of exported particulate organic matter is consistently above Redfield, implying that biogeochemical models based on Redfield stoichiometry underestimate POC export in the ocean. Potential POC export in the European Arctic Ocean is 10–30% higher when accounting for the variation in exported POC:PON compared to estimates based on constant stoichiometry. This study further shows that the exported POC:PON ratio varies among nitrogen-limited systems depending on new production, reflecting the system’s trophic state. Hence, the error induced by constant stoichiometry is largest in nutrient-poor oligotrophic systems, which represent the major part of the world’s oceans. Scaling export stoichiometry to trophic state improves the representation of the biological carbon pump in models, and more realistic POC export estimates are thus achieved. This is essential in order to correctly predict pathways of primary production in marine ecosystems, particularly in response to changes in inorganic nutrient supply and productivity.
